# Decreasing Preoperative Anxiety in Patients with Newly Available Multimodal Approaches—A Narrative Review

**DOI:** 10.3390/jcm14092940

**Published:** 2025-04-24

**Authors:** Weronika Kisielewska, Michał Kościółek, Weronika Kowalczyk, Bernard Mitura, Lidia Mitura, Sylwester Rogula, Piotr Konrad Leszczyński, Katarzyna Antosik, Kryspin Mitura

**Affiliations:** 1 Faculty of Medical and Health Sciences, University in Siedlce, 08-110 Siedlce, Polandchirurgia.siedlce@gmail.com (K.M.); 2 Third Department of Psychiatry, Institute of Psychiatry and Neurology, 02-957 Warsaw, Poland; 3 Faculty of Medicine, Medical University of Warsaw, 02-109 Warsaw, Poland; 4 Faculty of Medicine, Jagiellonian University Medical College, 31-008 Krakow, Poland; 5 Faculty of Medicine, Medical University of Lublin, 20-059 Lublin, Poland; 6 1st Chair and Department of Cardiology, Medical University of Warsaw, 02-097 Warsaw, Poland; 7 Department of General Surgery, Hospital in Siedlce, 08-110 Siedlce, Poland

**Keywords:** anxiety, anti-anxiety agents, informed consent, perioperative care, psychotherapy

## Abstract

Preoperative anxiety affects approximately 80% of adult patients; thus, identifying patients with excessive anxiety and implementing appropriate interventions can significantly reduce the risk of deterioration during the perioperative period. This narrative review presents current knowledge about pharmacological and nonpharmacological methods for reducing preoperative anxiety. Commonly used pharmacological options include benzodiazepines, ketamine, or fentanyl. Antidepressants have also been shown to be effective in alleviating symptoms, but they typically require four weeks to take effect. Establishment of supportive relationships with medical staff to help patients express their feelings have been shown to have a positive impact on anxiety reduction. Other nonpharmacological methods include the provision of information through informed consent forms, video materials, virtual reality, or the use of psychotherapeutic interventions such as breathing techniques, music therapy, or cognitive–behavioural therapy. Some studies suggest that essential oils may have a role in reducing perioperative anxiety. Nonpharmacological interventions can be used in patients of different ages. An increasing number of researchers advocate for a holistic approach that integrates less invasive and cost-effective interventions with conventional medicine. While various interventions have been proposed to manage preoperative anxiety, more research is needed to establish the most effective and feasible interventions for different patient populations.

## 1. Introduction

**Figure 1:**
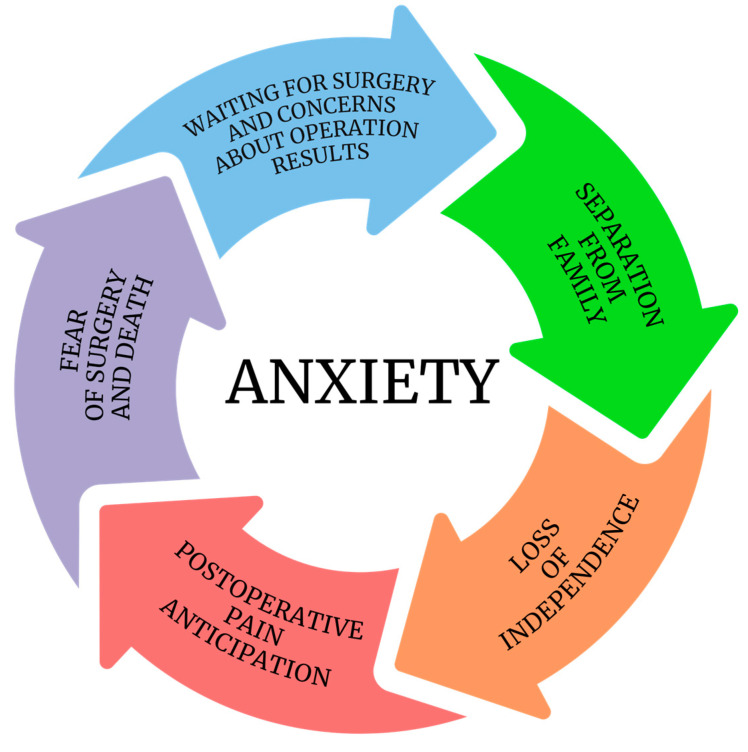
Development of perioperative anxiety [7].

Anxiety is a physiological state characterized by apprehension of a potential danger or misfortune that might occur in the future. Some degree of anxiety is a natural reaction to unpredictable and potentially threatening situations typical of the preoperative period. However, excessive anxiety may disrupt the postoperative course, causing an increased number of complications, such as nausea, vomiting, and impaired wound healing. Additionally, excessive anxiety may increase the intensity of postoperative pain and prolong the duration of hospitalization [1]. It is assumed that reducing anxiety may contribute to improving a patient’s cognitive ability and have a positive effect on the processes of understanding and remembering postoperative recommendations [2]. Notably, statistics indicate that 80% of adult patients experience preoperative anxiety, underscoring the prevalence and significance of this issue in the healthcare landscape [3]. The incidence of anxiety before the procedure depends mainly on age, sex, level of education, previous experience, marital status, and the type of surgery and anesthesia [4]. The most common causes of preoperative anxiety include waiting for surgery, concern about surgical outcomes, separation from family members, the anticipation of postoperative pain, the loss of independence, and the fear of surgery and death (Figure 1) [5]. Excessive anxiety before surgery is considered to constitute a heavy financial burden on the health care system [6]. These costs are believed to be the result of prolonged hospital stays and increased medication requirements. Therefore, reducing preoperative anxiety appears to be essential. Currently available methods used to mitigate anxiety before surgery can be classified as pharmacological and nonpharmacological methods. The former involves the administration of sedative and anti-anxiety drugs, whereas the latter involves education and relaxation techniques. Numerous studies have confirmed that appropriate intervention or treatment for preoperative anxiety may contribute to reducing the incidence of postoperative adverse events. The aim of our review was to systematize and present current pharmacological and nonpharmacological methods for reducing preoperative anxiety.

## 2. Methodology

This study was performed as a narrative literature review, aimed to comprehensively gather and summarize the available information on the pharmacologic and nonpharmacologic treatments for preoperative anxiety. Relevant articles were identified through PubMed and Google Scholar search engines using the key terms [informed consent or psychotherapy or verbal communication or educational videos or music or aromatherapy or massage or pharmacology] AND anxiety AND surgery. The search included articles published in English over the last five years. Exclusion criteria were as follows: reviews, meta-analyses, books, documents, and publications not written in English. After analyzing the articles, we presented the most important information on the following methods: informed consent process, psychotherapy, verbal communication, educational videos, music therapy, aromatherapy, massage, and pharmacological intervention.

The information on pharmacological interventions was supplemented by academic knowledge derived from established guidelines and reference materials, as well as the expertise of the authors.

## 3. Pharmacologic and Nonpharmacologic Treatments for Preoperative Anxiety

A total of 76 studies was selected for inclusion in this review, providing insights into approaches for managing preoperative anxiety. These works examined the effectiveness of both pharmacologic and nonpharmacologic interventions. To ensure clarity, the findings were organized into subsections that address each method in detail.

### 3.1. Informed Consent

We would like to draw attention to the fact that informed consent not only consists of signing a document but is a comprehensive process. It includes contact with a doctor, patient education, providing information about surgical procedures, and giving patients the opportunity to ask questions. The obtained data indicate that patients’ anxiety levels may be decreased by completing the informed consent process before surgical procedures. It is well known that a lack of information or insufficient information about medical interventions is associated with increased levels of anxiety. Interestingly, insufficient information and also excess information may contribute to increased patient anxiety [8]. These discrepancies can be explained by disparate patient attitudes towards obtaining information. Vigilant patients experience lower levels of anxiety if they are given as much information as possible, whereas avoidant patients reject any information that might cause distress [9]. These patient types must be considered when planning subsequent studies and selecting groups accordingly. Despite differences in preferences, research indicates that patients want to be informed and aware of possible risks. In view of patients’ willingness to be informed, several studies have been conducted to assess the impact of the format of informed consent on patients’ knowledge and comprehension. Some studies have demonstrated that incorporating video material into the consent process resulted in a better understanding of the proposed procedure and improved patient knowledge [10,11]. In contrast, Delcambre et al. reported that video supplementation during the consent process for Mohs micrographic surgery (MMS) was not associated with increased knowledge and comprehension rates [12]. However, compared with the control group, the intervention group had improved recognition of MMS-related risks and comprehension of received education materials. In another study, videos and written and oral informed consent were equivalent with respect to their effects on patient knowledge and comprehension [8].

There is ample evidence that the way in which information is presented may also have a direct effect on preoperative anxiety. Torres-Lagares et al. conducted research aiming to evaluate the effects of three formats of informed consent (written, oral, and video) on alleviating anxiety before surgery [9]. Both the oral and written formats were shown to reduce stress to some extent, whereas the video recording increased anxiety. Another study revealed that each of these informed consent modalities contributed to reducing preoperative anxiety, and the differences between the modalities were not statistically significant [8].

The use of multimedia supplements to the verbal informed consent process can reduce the amount of time a doctor has to devote to patients before a procedure. This is particularly important given the current limitations in time and human resources. However, it should be emphasized that neither video nor written informed consent processes can replace conversations between patients and physicians. There is evidence that the informed consent process is often inclusive and, to a large extent, depends on medical practitioners. This may result in numerous complications resulting from insufficient information provided to patients and may constitute grounds for criminal prosecution [13]. Written and video-informed consent formats enable standardization and unification of the information provided. This ensures that each patient receives the same information in an unbiased manner and reduces the risk of allegations. Unfortunately, there is evidence that more than half of patients do not read consent forms before signing them. This indicates the need to implement appropriate measures aimed at encouraging patients to become thoroughly familiar with the content of the consent form for surgery.

### 3.2. Psychotherapy

Psychotherapeutic interventions such as cognitive–behavioural therapy (CBT) can effectively reduce preoperative anxiety. Sessions may be conducted individually or in groups, aim to help patients cope with stress, alter their thinking and behaviour, and improve their overall mental well-being. As every person is unique, psychotherapy should be personalized to address preoperative anxiety in patients effectively. CBT is considered the gold standard for treating anxiety disorders [14]. A randomized controlled trial revealed that a 10-week CBT intervention before bariatric surgery decreased preoperative anxiety and depression symptoms [15]. CBT aims to identify and change destructive patterns that have a negative influence on patients’ behaviour and emotions. CBT encompasses a range of techniques and approaches that are designed to target maladaptive thoughts and behaviours. Diaphragmatic breathing [16], progressive muscle relaxation (PMR) [17], and guided imagery (GI) [18] are coping skills used within the CBT framework that promote relaxation. These techniques have been shown to be effective in reducing preoperative anxiety. GI is a type of cognitive therapy which involves recalling or reconstructing sensory and emotional experiences. Patients are encouraged to recreate mental images during times of high distress. The effectiveness of GI in reducing anxiety before various types of surgical procedures, such as laparoscopic cholecystectomy [18], orthopaedic surgery [19], and dental procedures [20], has been confirmed. Additionally, research conducted by Aker et al. revealed that GI decreased anxiety among women before caesarean section [21]. As a nonpharmacological method, GI enables the avoidance of the use of medications that may pose a risk to the foetus. Diaphragmatic breathing is a deep, controlled breathing exercise that fully engages the diaphragm. The results of a randomized, controlled, semiexperimental study indicated that the incorporation of breathing exercises may yield positive outcomes by decreasing anxiety levels and pain scores [16]. PMR is a form of therapy that involves tightening and relaxing muscle groups [22]. In a study conducted by Uysal et al. on 38 patients diagnosed with aneurysms, PMR not only reduced anxiety before surgery but also decreased blood pressure and heart rate [23]. These are particularly important results, as there is evidence that lowering blood pressure may reduce the risk of aneurysm rupture [24].

Distraction is another practical cognitive coping skill that encourages patients to shift their focus from distressing or threatening aspects of the perioperative process to more calming and pleasant activities or thoughts. Distraction techniques can be distinguished into active and passive forms. Active distraction promotes patient engagement and involves video games and virtual reality (VR), whereas passive distraction requires only the ability to observe the distracting stimulus and includes watching videos and listening to music [25]. The degree of patient involvement in the distraction procedure is highly important in reducing anxiety. Tordet et al. revealed that playing video games, as a more engaging method, was more effective than watching a film [26]. These findings are in accordance with those of another study carried out by Yamashita et al. [27]. Similarly, Pandrangi et al. did not find a significant difference between two active distraction methods, namely, gaming and mindfulness, in reducing perioperative stress [28].

Immersive VR is gaining popularity as a sophisticated technique for reducing anxiety during the perioperative period [29]. As an interactive form of distraction, VR allows for complete immersion in a created three-dimensional space that stimulates a user’s senses, such as sight and hearing. To date, the exact mechanism by which VR relieves anxiety is still poorly understood. However, it is assumed that distracting a patient’s attention results in a decrease in cognitive and concentration abilities and consequently interrupts the pathological thought process [29]. Another possible mechanism of the anxiolytic effect is modulation and consequently reduced activity in anxiety-related areas, as seen in functional magnetic resonance imaging (fMRI) images. Almedhesh et al. reported interesting results concerning the beneficial effect of VR on reducing anxiety during caesarean section [30]. These findings are essential because of the limited opportunities for the use of pharmacological agents in pregnant women. VR, as a low-risk method, seems to be a great alternative for reducing anxiety during caesarean section, especially since caesarean section is performed under regional anaesthesia. In contrast to anxiolytics, VR enables a conscious experience of childbirth and does not pose a threat to the foetus. The positive effect of VR on patients undergoing surgeries under regional anaesthesia has been confirmed in other studies [30,31]. In addition to its direct anxiolytic effect, VR decreases anxiety by isolating patients from ambient noise in the operating room, which is especially important during regional anesthesia as patients are awake and conscious. VR can help reduce the use of sedative and pain-alleviating drugs. Faruki et al. reported that VR immersion helped reduce propofol dosing without affecting overall satisfaction, pain scores, or postoperative functional outcomes [32]. Another study demonstrated that the incorporation of VR resulted in marked reductions in the amount of midazolam used intraoperatively [33]. Moreover, in the same study, the use of VR was associated with greater patient satisfaction. Additionally, Tharion et al. [34] reported that the postoperative satisfaction score in the VR group was significantly higher than that in the control group receiving midazolam, which confirms the findings obtained by Gamal et al. [33]. These results indicate that VR may be an alternative to sedation for many anxiety-provoking surgical procedures. Some studies have illustrated the impact of VR on physiological parameters. However, the exact mechanism has not yet been studied. In a group of patients undergoing abdominal wall surgery, the use of VR was associated with a reduction in systolic and diastolic blood pressure, heart rate, and respiratory rate. It was also correlated with increased saturation values. Moreover, Ghobadi et al. demonstrated that the incorporation of VR resulted in decreased muscle tension, pain, and fatigue according to electromyography (EMG) parameters; and lower heart rates, sympathetic nervous system activity, pain, and anxiety according to electrocardiogram (ECG) and skin conductance response [35].

In contrast to the studies mentioned above, Gurbuz et al. did not observe any effect of VR on reducing perioperative anxiety [36]. This highlights the need for further research to confirm the benefit of VR during the perioperative period. Conclusively, the available data indicate the significant potential of psychotherapy to reduce perioperative anxiety. Unfortunately, owing to the limited number of psychotherapists, access to psychotherapy is limited, which significantly hinders its use in reducing perioperative anxiety, even though it is safe and inexpensive.

### 3.3. Educational Videos

The use of video education has gained increasing attention as a method to reduce preoperative anxiety in patients. This approach ensures that all patients receive the same standardized information about planned procedures, minimizing the risk of insufficient or overly detailed explanations. Educational videos ensure that all patients receive consistent, accurate information about their surgical procedures, which improves their understanding of procedural risks compared with receiving only verbal explanations [12,37]. In addition, video education reduces the workload of healthcare workers. Previous studies reported that patients (including paediatric patients) who watched an educational video needed less time with an anaesthesiologist while maintaining comparable levels of satisfaction and understanding [38,39]. Reducing patient anxiety is a primary goal of preoperative education, and video education has been consistently effective in achieving this goal compared with verbal education alone [40,41]. Educational videos also save valuable time for physicians by addressing common patient concerns preemptively. These videos reduce unplanned healthcare use, improve recovery outcomes [42], and enhance patient and family satisfaction [43]. In paediatric patients, videos reduce preoperative anxiety and improve compliance during anaesthesia induction, resulting in smoother procedural workflows [44,45]. These findings highlight how videos not only improve patient outcomes but also enhance patient satisfaction by addressing their concerns comprehensively. The benefit of videos extends to caregivers as well. Mothers of children undergoing surgery reported reduced anxiety when provided with an educational video supplementing standard care [46,47]. Innovative approaches such as 360-degree VR tours in operating rooms have further improved anxiety reduction by immersing patients in the surgical environment and increasing their readiness [48,49,50].

Despite their broad success, educational videos do not always lead to significant reductions in anxiety. For example, a study on oculoplastic surgery revealed that while videos increased awareness of postoperative outcomes, they did not significantly affect anxiety levels [51]. Similarly, a trial on third molar extractions suggested that videos might inadvertently increase anxiety when viewed without proper contextualization by healthcare providers [52]. These discrepancies underscore the importance of tailoring video content to the specific needs and characteristics of patient populations.

Compared with standard education, interactive and multimedia-based educational tools, such as comic booklets and games, are associated with lower anxiety levels and increased compliance and represent a promising evolution of traditional videos [53,54]. However, the passive learning technique of watching a movie to reduce anxiety levels is not effective, but active distraction through video games has been shown to be a valuable method for reducing preoperative anxiety [1]. Peer-based video education, which involves patient reviews, has also been shown to be particularly effective in reducing preoperative anxiety [55]. Additionally, integrating videos into broader protocols, such as enhanced recovery after surgery (ERAS), significantly improved postoperative outcomes, including reduced complications and faster recovery [56]. These innovative applications demonstrate the adaptability of educational videos to diverse clinical contexts.

VR methods are playing an increasingly important role in medicine. VR can be used for operating room simulations and can help reduce anxiety, especially for patients who feel uncomfortable witnessing medical procedures, but it is not effective for all patients. These tools facilitate communication with patients after they have watched a video or simulation, allowing for more effective discussions and questions. However, despite its advantages, VR cannot replace basic doctor–patient relationships.

### 3.4. Verbal Communication

Verbal communication with patients is a key element of professional medical care. Doctor–patient relationships based on mutual respect and trust have a considerable effect on the effects of treatment. A patient who trusts their doctor feels safer and is more willing to follow medical recommendations [57]. The evidence from previous studies indicates that properly conducted conversations may contribute to reducing anxiety in patients undergoing surgery [58]. Favourable doctor–patient relationships involve several essential elements, such as trust, empathy, respect, and compassion. Anxiety often stems from being unfamiliar with the environment and being unclear about what to expect. Healthcare professionals should introduce themselves and inform patients about what they are going to do and for what reason. When meeting an anxious patient, healthcare professionals should focus on reassurance and empathy. Demonstrating understanding and assuring support are also important [58]. Additionally, medical staff should avoid the use of medical jargon and common medical abbreviations. Speaking in an incomprehensible manner may impede physician–patient relationships, and negatively impact patient outcomes. Safe relationships help patients identify the cause of anxiety and encourages them to express their feelings. Therefore, it is worth making every effort to ensure that communication with a patient allows both parties to feel heard and understood.

### 3.5. Music Therapy

The available data indicate a significant impact of music interventions on reducing perioperative anxiety. Evidence shows that listening to music not only reduces anxiety but also relieves pain [59,60,61,62,63], reduces the risk of postoperative nausea and vomiting (PONV) [59,63] and increases patient satisfaction [64,65,66]. Thus, music seems be an alternative or complement to pharmacological approaches. Some studies have reported that music interventions reduce sedative requirements in patients undergoing surgery under general [59] and regional anaesthesia [67,68]. Corresponding results were obtained in two studies, which showed that the music group required less propofol to maintain an adequate level of sedation, as measured by the bispectral index (BIS) [59,64]. Interestingly, Koelsch et al. revealed lower propofol consumption in the music group. Despite this, the mean BIS value was nominally lower in the music group than in the control group, reflecting a deeper level of sedation [67]. Notably, some studies seem to confirm these results by showing the sedative potential of music interventions. Giordano et al. [65] reported that patients in the music therapy group had significantly lower BIS values than did patients in the intravenous midazolam group. Research also suggests that both interventions had the same effect on sympathetic nervous system activity and the hormone response, as there were no significant differences in PRL, GH, or cortisol levels after the surgical procedure. Moreover, patients who listened to music reported higher levels of satisfaction [65]. Gökcek et al. reported that the incidence of intraoperative awareness was lower in the music group than in the control group [69]. Additionally, on the basis of the Riker Sedation–Agitation Scale (RSAS), the subjects in the music group had a better awakening quality [69]. Music therapy seems to be a promising option for complementing sedative treatment and consequently reducing the potential side effects associated with the use of anaesthetics. However, no study has indicated that music interventions can completely replace anxiolytic medications [65]. Although the underlying mechanism of music therapy remains unclear, listening to music may decrease the activity of the sympathetic nervous system, contributing to a reduction in somatic symptoms of anxiety, such as increased heart rate, respiratory rate, blood pressure [70,71], and pupil size [70]. Moreover, music exerts its anxiolytic effect through its inhibitory effect on the amygdala, which plays a key role in anxiety development [72]. Music influences brain regions responsible for anxiety, such as the prefrontal area, rostral ventromedial medulla, and periaqueductal grey matter. It also relieves pain, as these structures are part of the descending pain modulatory system [65]. One of the most studied topics among recent studies is the influence of patient preferences and type of music on the degree of anxiety reduction. Some studies have shown a significant effect of patients’ self-selected favourite music on reducing preoperative anxiety [71]. In contrast to these results, another study showed that preselected music contributed to a reduction in preoperative anxiety, whereas self-selected music did not [73]. Reynaud et al. reported that regardless of whether patients chose the music themselves or listened to predetermined music, anxiety reduction occurred, and the differences between the groups were not significant [74]. Additionally, Uğraş et al. compared the efficacy of three genres of music and reported that classical Western music was significantly more effective than Turkish music [75]. However, the work of Pellicer et al. did not indicate a predominance of any type of music studied [76]. Regarding the abovementioned results, further studies that focus on determining whether the effects of music interventions differ on the basis of the type of music would enhance the knowledge base on this topic. Moreover, a randomized controlled study revealed that the degree of anxiety reduction also depends on the sound presentation technique used [77]. Binaural beat audio created with the dynamic spectrum phase shift (DMSPS) algorithm was shown to be more effective than normal music [77]. In addition to the general effect of music on reducing preoperative anxiety, it has been shown that the use of headphones additionally enhances the anxiolytic effect as they muffle sounds from the operating room. It is assumed that unfamiliar sounds such as conversations with medical staff are independent factors that increase anxiety in patients. Surprising results were also obtained in a study conducted by Demirci et al., which demonstrated that listening to music alone is more effective in managing preoperative anxiety than the combination of listening to music and watching a movie, as it seems that the combination of two methods would contribute to a more significant reduction in anxiety than would a single method [78]. The authors assumed that one of the possible reasons was the inability of patients to select video material, while they could listen to their favourite music. Nevertheless, these findings highlight the efficacy of both interventions in addressing anxiety even when patients are not able to make video selections. Notably, music has been shown to have a positive effect on reducing anxiety in patients undergoing surgery under spinal [79,80] or regional anaesthesia [60,61,70,78]. These types of surgical procedures cause particularly high levels of anxiety, as patients are awake and exposed to various types of audiovisual stimuli [79]. Notably, music not only stabilized haemodynamic parameters but also had a sedative effect [79]. Thus, music should be considered a complement to pharmacological methods for relieving anxiety during surgical procedures when patients are fully conscious. A positive effect of music on relieving anxiety before coronary artery bypass grafting (CABG) was also observed, which, similar to surgery under regional or spinal anaesthesia, is a procedure associated with a high level of anxiety. Finally, the results of the collected studies indicate that music interventions are effective in reducing perioperative anxiety among children [63,81]. In addition, lower anxiety levels improved communication skills and contributed to better cooperation with medical personnel. Notably, some studies have not shown a beneficial effect of music on the reduction of perioperative anxiety [73,82,83]. Accordingly, further randomized controlled trials are necessary to confirm the validity of the collected findings and elucidate the observed discrepancies. In conclusion, most of the data indicate that listening to music in the perioperative period is an effective, inexpensive, and safe method. For this reason, it should be increasingly introduced into everyday clinical practice.

### 3.6. Aromatherapy

Aromatherapy involves the therapeutic use of essential oils to enhance health and well-being. Common aromatherapy oils known to reduce anxiety include lavender (*Lavandula angustifolia*), rose (*Rosa damascena*), bergamot (*Citrus aurantium*), orange (*Citrus sinensis*), lemon (*Citrus limon*), sandalwood (*Santalum album*), clary sage (*Salvia sclarea*), Roman chamomile (*Anthemis nobilis*), and rose-scented geranium (*Pelargonium* spp.) [84]. Essential oils may be used in several different ways, such as inhalation, massage, bath, compression, and application to the skin surface [85,86]. These oils exhibit holistic properties and were reported to have significant antiseptic, antibacterial, antiviral, antiparasitic, and antifungal effects. Their anti-inflammatory, antinociceptive and anticancer effects should also be emphasized [87]. Aromatherapy may improve mental well-being and alleviate nausea, vomiting [88], and sleep disturbances [89]. Several clinical studies have confirmed that essential oils may reduce perioperative anxiety. They affect the limbic system and amygdala and stimulate the release of neurotransmitters such as serotonin and endorphins, which have anxiolytic and mood-boosting effects [90]. It is also believed that the compounds contained in essential oils may exert their effects through the gamma-aminobutyric acid (GABA) neurotransmitter and affect the parasympathetic system [91]. The available data reveal the wide use of aromatherapy and its positive anxiolytic effect in patients undergoing various types of surgical interventions, including percutaneous nephrolithotomy [92], breast cancer surgery [91], abdominal surgery [93], otolaryngology, [94] and dental procedures [90,95]. A study conducted by Rahman et al. revealed that the use of lavender oil, in addition to reducing anxiety, contributed to reducing the amount of propofol required for the induction of anaesthesia [96]. This indicates the potential use of aromatherapy as a complement to premedication in the future. Essential oils, due to their simplicity and ease of use, offer an effective way to alleviate anxiety in patients undergoing outpatient surgical procedures. These procedures are characterized by a short waiting time for surgery and require a convenient method for anxiety reduction [94]. Notably, owing to its safety profile and low risk of side effects, aromatherapy is widely used in children, and evidence regarding the use of aromatherapy in infants is increasing [97]. Interesting results were obtained in two studies conducted by Nirmala et al. and Abdalhai et al., which revealed a positive effect of aromatherapy on reducing anxiety in children before a dental procedure [90,95]. It is assumed that the aroma of the oils alleviates the odour originating from the dentist’s office, which is one of the factors that increases the feeling of anxiety before dental procedures [90]. The results of this study are extremely important, as reducing children’s fear of dental procedures is crucial to maintaining their oral health.

These studies suggest that the type of oil may have an effect on the degree of anxiety reduction. However, the evidence is not robust, and more studies should be carried out. A randomized, three-group study of 90 women undergoing caesarean section revealed that Damask oil was more effective than lavender oil in reducing postoperative anxiety. Additionally, the mean pain score in the Damask group was lower than that in the Lavender group [98]. Similarly, Bahrami et al. reported that patients who were awaiting emergency orthopaedic surgery and who received Damask oil had lower anxiety levels than did those who received chamomile oil [99].

While essential oils have numerous potential health benefits, it is crucial to use them with caution, as they also carry the risk of adverse events. The most common hypersensitivity reactions can be avoided by diluting the essential oils and, as a result, reducing the concentration of their active substances [86]. The type of oil, its concentration, and method of administration should be adapted to the individual profile of a patient. Particular caution should be exercised for pregnant and breastfeeding women, children, people with skin damage, those with hypertension, those with lung diseases, those who are undergoing oncological treatment and those with epilepsy [86]. Unfortunately, some of the conducted studies did not show a positive effect of aromatherapy on reducing preoperative stress [100,101]. A single-blind clinical trial assessing the effect of inhaling three drops of 4% rose oil on reducing anxiety in patients before CABG did not reveal a significant effect. In contrast, another study revealed that the inhalation of 12% rose oil significantly reduced preoperative anxiety before cholecystectomy. The discrepancies may result from varying oil concentrations used in the studies, inhalation duration, number of participants and anxiety assessment methods. Additionally, the initial level of anxiety, which is particularly high in the case of patients undergoing heart procedures, may have an impact. We aimed to provide valuable insight into aromatherapy, emphasizing its potential as a natural and potent remedy for reducing perioperative anxiety. Notably, this is a cost-effective, convenient method with a low side effect profile. It may serve as an alternative or complement to other methods for managing anxiety before surgery [96].

### 3.7. Massage

Massage is an intervention that has also been shown to be effective in reducing preoperative anxiety. The positive anxiolytic effect has been reported in patients undergoing various types of surgical procedures, such as dental surgery [102], cataract surgery [103], breast cancer surgery [104] and cervical spine surgery [105]. The current evidence shows that massage therapy is also effective as a nonpharmacological tool for decreasing postsurgical pain [105]. Massage involves the manual or mechanical performance of various touch techniques that affect human tissues. It is believed that the anxiolytic effect of this method results from the activation of the parasympathetic component and the reduction in the amount of cortisol released. Massage serves as an essential therapeutic intervention in nursing care that is easy to learn and cost-effective. However, owing to the simplicity of massage, not only nurses but also family members can learn the technique and perform it even at home [106]. Massage therapy has been shown to be an effective method for reducing anxiety in patients awaiting ambulatory surgery and outpatient procedures [107,108]. Interesting results were obtained in a study conducted by Ni et al. [107], which revealed the beneficial effect of machine-based hand massage on reducing anxiety in patients undergoing various types of ambulatory operations. The use of mechanical devices reduces the workload of the person performing the procedure and consequently makes it more convenient to use [107]. This feature is particularly important in outpatient care, which requires noncomplex anxiety-reducing interventions. Although the study revealed that massage alleviated anxiety, it had no significant effect on vital parameters. In contrast, a clinical study evaluating the effect of massage on reducing anxiety in patients undergoing cataract surgery revealed significant effects of massage on reducing blood pressure, heart rate and respiratory rate [103]. Another study conducted by Farahani et al. reported corresponding results [109]. Considering the discrepancies among studies, further research on the relationship between massage and vital signs is needed.

In addition to the methods used to assess the overall impact of massage on reducing anxiety, two massage techniques, i.e., hand and foot massage, were compared in terms of their beneficial effects on reducing anxiety, and no significant differences were detected on the basis of visual analogue scale scores and physiological indicators [109]. To date, data on the effects of foot massage on anxiety reduction are limited. However, this topic is gaining more popularity. Foot massage is a suitable method for reducing anxiety in patients after cervical spine surgery and is an alternative to classic back massage, as it does not pose a risk of spinal cord injury [105].

Moreover, the application of foot massage seems to have an effect on kinesiophobia, balance, daily living skills, mobility, and mental health among older patients in the postoperative period. Improving these parameters can effectively shorten the duration of hospitalization and reduce the risk of readmission to the hospital [110]. The results of some studies indicate that the addition of massage to aromatherapy enhances its anxiolytic effect [111]. However, counterintuitively, combining massage with acupuncture has not been shown to be more effective than massage alone [104]. To summarize, massage therapy is a cost-effective, noninvasive and safe method for reducing anxiety. However, there are situations in which its use is not advisable. Massage is not recommended for people with severe immune disorders, hypersensitivity to touch, allergies to oils, acute coronary syndrome, pregnancy, dermatological diseases, neuropathy, and delirium.

### 3.8. Pharmacology Approach

Currently, pharmacological methods are commonly used to reduce preoperative anxiety. Benzodiazepines, ketamine, or fentanyl are often administered [112]. Unfortunately, the administration of medications is associated with the risk of side effects, including hypotension or cardiac arrhythmias, respiratory depression, gastrointestinal dysmotility, nausea, vomiting, drowsiness, or impaired vision [113,114,115,116,117,118]. Moreover, drugs can interact with anaesthetic agents to prolong the recovery phase and duration of hospitalization [119].

The choice of medication resides with the anaesthesiologist depending on the individual patient profile. Benzodiazepines are the first-line drugs used to reduce preoperative anxiety [120]. They are characterized by a broad action profile and have anxiolytic, sedative, amnestic, muscle relaxing, and anticonvulsant effects [119]. One significant factor that limits the administration of these drugs before surgical procedures is their potential to cause anterograde amnesia, which involves the inability to remember events after taking the medication [121]. Although there are no absolute contraindications to the use of benzodiazepines, except for a history of severe hypersensitivity reactions, special caution should be exercised in patients with a history of addiction or sleep apnoea [122,123]. Those with liver or kidney failure require particular attention because benzodiazepines have different excretion mechanisms. Benzodiazepines with a short half-life, such as lorazepam, are often used for premedication when administered orally. In the operating room, short-acting intravenous benzodiazepines like midazolam are frequently used [119].

Overall, the drugs most commonly used to reduce anxiety disorders are selective serotonin reuptake inhibitors (SSRIs) and serotonin-norepinephrine reuptake inhibitors (SNRIs) [124]. They are most effective in alleviating symptoms and preventing relapses and have a good safety profile. However, the limitation in the use of SSRIs and SNRIs for preoperative stress is the time at which they begin to work; their effect becomes apparent after approximately three weeks of use [125].

In contrast to SSRIs and SNRIs, benzodiazepines have varying durations of action, with some taking effect within 30 min, so they can be administered shortly before a procedure [113]. Nonetheless, owing to their addictive potential, short-acting benzodiazepines especially should be taken for only a short period [126]. It is also worth emphasizing the remaining concern among patients regarding the use of psychotropic drugs. Most patients are afraid of dependency and potential side effects. Hence, there is a growing demand for alternative or complementary methods to reduce anxiety. Finally, importantly, pharmacological treatment should be prescribed and monitored by healthcare professionals, considering individual patient characteristics, potential side effects and contraindications. A detailed characterization of individual medications is outside the scope of this review.

## 4. Discussion

**Figure 2:**
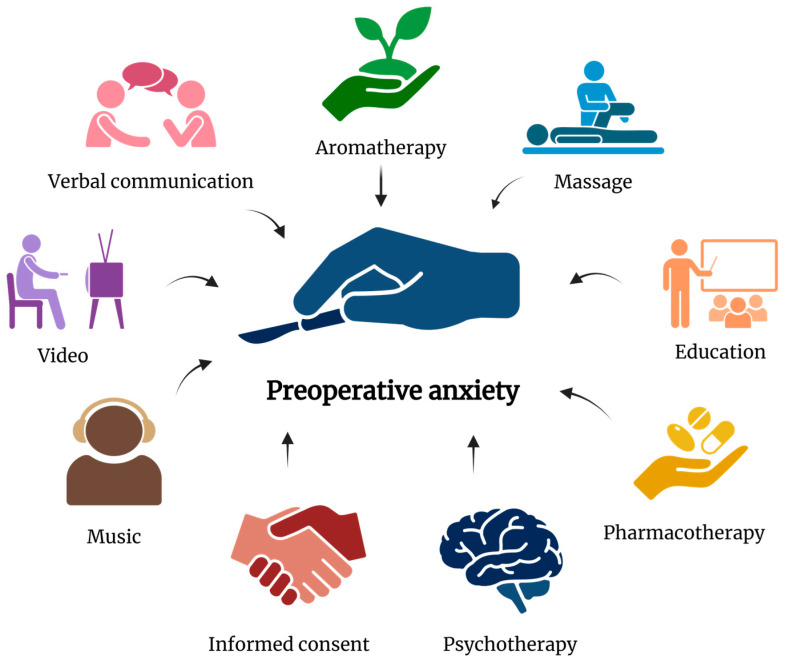
Pharmacological and nonpharmacological methods for reducing perioperative anxiety [128].

The above literature review revealed a wide range of different types of interventions and promising results (Figure 2). Currently, the most common method for reducing anxiety is administering anxiolytic medication. The administration of anxiolytic and hypnotic drugs, despite their unquestionable effectiveness, is associated with many side effects. In recent years, nonpharmacological approaches have attracted increasing interest in research. Researchers increasingly advocate for a holistic approach, incorporating less invasive and costly interventions as a complement or alternative to conventional medicine. Owing to their safety profile, nonpharmacological interventions can be used in patients of different ages who are undergoing various surgical procedures. Agbayani et al. described nonpharmacological approaches as effective, low-risk options. These interventions not only provide psychological comfort but also minimize the need for sedative medication, reducing the overall risk of side effects [127]. Considering their profitability and relatively low-risk profile, nonpharmacological interventions are excellent alternatives to the incorporation of pharmacological agents.

Nevertheless, there are several factors that constitute limitations for their widespread use, such as the availability of such interventions and well-qualified personnel. Although CBT is one of the most effective methods for treating anxiety disorders, limited access to specialists still poses a challenge. Koffel et al. emphasized that logistical barriers, such as insufficient trained personnel, limit the broader implementation of evidence-based psychological treatments like CBT [129]. Telemedicine may help overcome these challenges, but further development is needed. It is worth mentioning the enormous role of nurses in reducing preoperative anxiety. Nurses maintain continuous contact with patients and, through various activities, may significantly contribute to reducing anxiety and stress. Nursing care includes providing psychological support, implementing relaxation techniques, and providing information about procedures and potential complications. The available data show a wide range of possibilities for reducing perioperative anxiety, which enables the selection of the most appropriate method regarding different patient profiles and types of surgery. The results of most studies revealed a positive impact of pharmacological and nonpharmacological approaches on the reduction of anxiety in patients who are candidates for surgery. In contrast to these findings, some studies have not confirmed the beneficial effects of the applied methods. Accordingly, further research is needed to improve the quality and effectiveness of the presented methods.

## 5. Conclusions

Preoperative anxiety can have a profound impact on surgical treatment outcomes. This review highlights the great need to mitigate anxiety in patients undergoing surgery. Properly identifying patients with excessive anxiety and implementing appropriate interventions can significantly alleviate anxiety and reduce its consequences. We hope that this article will increase the awareness of healthcare professionals, thereby improving quality of care and leading to better treatment outcomes.

## 6. Future Perspectives and Directions

The above literature review highlights the enormous progress that has been made in the development of various nonpharmacological methods aimed at reducing preoperative anxiety in the last decade. The conducted studies indicate the prodigious potential of these interventions in reducing fear of surgical procedures. Although numerous investigations have been conducted, there is still little information about their physiological mechanisms of action. Furthermore, while various interventions have been proposed to manage preoperative anxiety, more research is needed to establish the most effective and feasible interventions for different patient populations. Further studies should also assess the long-term effects of the implemented methods. Some of the obtained data did not demonstrate a beneficial effect of the methods used for alleviating the fear of surgery. Thus, there is a need to conduct other randomized controlled trials to clarify these discrepancies and assess the effectiveness of these interventions.

## Data Availability

Source data are available upon request to the corresponding author.

## Abbreviations

The following abbreviations are used in this manuscript:

BIS	Bispectral index
CABG	Coronary artery bypass grafting
CBT	Cognitive-behavioural therapy
DMSPS	Dynamic spectrum phase shift
ECG	Electrocardiogram
EMG	Electromyography
ERAS	Enhanced recovery after surgery
fMRI	Functional magnetic resonance imaging
GABA	Gamma-aminobutyric acid
GH	Growth hormone
GI	Guided imagery
MMS	Mohs micrographic surgery
PMR	Progressive muscle relaxation
PONV	Postoperative nausea and vomiting
PRL	Prolactin
RSAS	Riker Sedation-Agitation Scale
SNRI	Serotonin-norepinephrine reuptake inhibitor
SSRI	Selective serotonin reuptake inhibitor
VR	Virtual reality

## References

[1] Gu X., Zhang Y., Wei W., Zhu J. (2023). Effects of Preoperative Anxiety on Postoperative Outcomes and Sleep Quality in Patients Undergoing Laparoscopic Gynecological Surgery. J. Clin. Med..

[2] Gilmartin J., Wright K. (2008). Day surgery: Patients’ felt abandoned during the preoperative wait. J. Clin. Nurs..

[3] Abate S.M., Chekol Y.A., Basu B. (2020). Global prevalence and determinants of preoperative anxiety among surgical patients: A systematic review and meta-analysis. Int. J. Surg. Open.

[4] Adhikari S.P., Pathak B.D., Ghimire B., Baniya S., Joshi P., Kafle P., Adhikari P., Rana A., Regmi L., Dhakal B. (2023). Prevalence of pre-operative anxiety and associated risk factors among patients awaiting elective surgery in a tertiary care hospital. F1000Research.

[5] Omkaram S., Reddy C.G., Murthy P.S., Chaudhury S. (2023). Prevalence of preoperative anxiety in patients posted for surgical procedures and its relation to the doses of anesthetic drugs: A cross-sectional study. Ind. Psychiatry J..

[6] Kiecolt-Glaser J.K., Page G.G., Marucha P.T., MacCallum R.C., Glaser R. (1998). Psychological influences on surgical recovery. Perspectives from psychoneuroimmunology. Am. Psychol..

[7] Rogula S. (2025). Development of Perioperative Anxiety. https://BioRender.com/k78v767.

[8] Goldberger J.J., Kruse J., Kadish A.H., Passman R., Bergner D.W. (2011). Effect of informed consent format on patient anxiety, knowledge, and satisfaction. Am. Heart J..

[9] Torres-Lagares D., Heras-Meseguer M., Azcárate-Velázquez F., Hita-Iglesias P., Ruiz-de-León-Hernández G., Hernández-Pacheco E., Gutiérrez-Pérez J.L. (2014). The effects of informed consent format on preoperative anxiety in patients undergoing inferior third molar surgery. Med. Oral Patol. Oral Y Cir. Bucal.

[10] Osterloh J., Müller-Seubert W., Cai A., Arkudas A., Horch R.R.E. (2024). Is there an impact of a video-based patient informed consent in elective hand surgery?. Arch. Orthop. Trauma Surg..

[11] Bremer K., Brown E., Schenkel R., Walters R.W., Nandipati K.C. (2024). Video consent significantly improves patient knowledge of general surgery procedures. Surg. Endosc..

[12] Delcambre M., Haynes D., Hajar T., Golden S., Bar A., Latour E., Leitenberger J.J. (2020). Using a Multimedia Tool for Informed Consent in Mohs Surgery: A Randomized Trial Measuring Effects on Patient Anxiety, Knowledge, and Satisfaction. Dermatol. Surg..

[13] Gogos A.J., Clark R.B., Bismark M.M., Gruen R.L., Studdert D.M. (2011). When informed consent goes poorly: A descriptive study of medical negligence claims and patient complaints. Med. J. Aust..

[14] Bhattacharya S., Goicoechea C., Heshmati S., Carpenter J.K., Hofmann S.G. (2023). Efficacy of Cognitive Behavioral Therapy for Anxiety-Related Disorders: A Meta-Analysis of Recent Literature. Curr. Psychiatry Rep..

[15] Gade H., Friborg O., Rosenvinge J.H., Småstuen M.C., Hjelmesæth J. (2015). The Impact of a Preoperative Cognitive Behavioural Therapy (CBT) on Dysfunctional Eating Behaviours, Affective Symptoms and Body Weight 1 Year after Bariatric Surgery: A Randomised Controlled Trial. Obes. Surg..

[16] Karagoz O., Sayilan A.A. (2023). The effection pain and anxiety of a breathing exercise applied following laparoscopic cholecystectomy: A randomized controlled study. Brain Behav. Immun. Integr..

[17] Aristiani I.D., Heri Susanti I. (2022). Management of Preoperative Anxiety Patients with Progressive Muscle Relaxation Therapy Interventions. Genius J..

[18] Lu Y.J., Lee M.C., Chen C.Y., Liang S.Y., Li Y.P., Chen H.M. (2022). Effect of Guided Imagery Meditation During Laparoscopic Cholecystectomy on Reducing Anxiety: A Randomized Controlled Trial. Pain Manag. Nurs..

[19] Gunes H., Saritas S., Ozdemir A., Bulbul A.S. (2023). The effect of guided imagery applied on geriatric orthopaedic patients on preoperative anxiety and comfort. ANZ J. Surg..

[20] Ko Y.C., Chou A.H., Wu C.F., Chen J., Chen C.Y. (2021). Using Guided Imagery to Relieve the Anxiety of Preschool Children Undergoing Dental Procedures. J. PeriAnesthesia Nurs..

[21] Aker M.N., Öner Cengiz H., Yilmaz Sezer N. (2024). The effect of guided imagery pre-cesarean section on the perceived preoperative anxiety, surgical fear, and physiological parameters of women: A randomized controlled trial. Eur. J. Integr. Med..

[22] LeRoy S., Elixson E.M., O’Brien P., Tong E., Turpin S., Uzark K. (2003). Recommendations for preparing children and adolescents for invasive cardiac procedures: A statement from the American Heart Association Pediatric Nursing Subcommittee of the Council on Cardiovascular Nursing in collaboration with the Council on Cardiovascular Diseases of the Young. Circulation.

[23] Uysal D.S.Ö., Demirgil B.T. (2023). The Effects of Progressive Muscle Relaxation on Preoperative Anxiety and Blood Pressure in Patient with Aneurysm. Nurs. Health Sci. J..

[24] Tada Y., Wada K., Shimada K., Makino H., Liang E.I., Murakami S., Kudo M., Kitazato K.T., Nagahiro S., Hashimoto T. (2014). Roles of hypertension in the rupture of intracranial aneurysms. Stroke.

[25] Pius L.E., Jacob L., Varghese S.P., Raju R., Mathew A., Raj J.M., Varghese E.S., John J.J. Comparing the Efficacy of Active and Passive Distraction for Reducing Pain and Fear During Intravenous Cannulation in Children: A Randomized Control Trial. https://socialinnovationsjournal.org/editions/issue-50/75-disruptive-innovations/2870-comparing-the-efficacy-of-active-and-passive-distraction-for-reducing-pain-and-fear-during-intravenous-cannulation-in-children-a-randomized-control-trial.

[26] Tordet C., Erhel S., Dodeler V., Gonthier C., Jamet E., Nardi N., Rouxel G., Wodey E. (2025). The benefits of experiencing flow through distracting activities: Flow reduces preoperative anxiety in children before surgery, but not postoperative difficulties. Psychol. Health.

[27] Yamashita Y., Aijima R., Danjo A. (2023). Clinical effects of different virtual reality presentation content on anxiety and pain: A randomized controlled trial. Sci. Rep..

[28] Pandrangi V.C., Low G., Slijepcevic A., Shah S., Shindo M., Schindler J., Colaianni A., Clayburgh D., Andersen P., Flint P. (2024). Use of Perioperative Virtual Reality Experiences on Anxiety and Pain: A Randomized Comparative Trial. Laryngoscope.

[29] Flores A., Hoffman H.G., Navarro-Haro M.V., Garcia-Palacios A., Atzori B., Le May S., Alhalabi W., Sampaio M., Fontenot M.R., Mason K.P. (2023). Using Immersive Virtual Reality Distraction to Reduce Fear and Anxiety before Surgery. Healthcare.

[30] Almedhesh S.A., Elgzar W.T., Ibrahim H.A., Osman H.A. (2022). The effect of virtual reality on anxiety, stress, and hemodynamic parameters during cesarean section: A randomized controlled clinical trial. Saudi Med. J..

[31] Turan A.Z., Yilmaz M., Saracoglu T. (2021). The effect of virtual reality glasses on anxiety during surgery under spinal anesthesia: A randomized controlled study. Anaesth. Pain Intensive Care.

[32] Faruki A.A., Nguyen T.B., Gasangwa D.V., Levy N., Proeschel S., Yu J., Ip V., McGourty M., Korsunsky G., Novack V. (2022). Virtual reality immersion compared to monitored anesthesia care for hand surgery: A randomized controlled trial. PLoS ONE.

[33] Gamal M., Rady A., Gamal M., Hassan H. (2023). Efficacy of virtual reality distraction technique for anxiety and pain control in orthopedic forearm surgeries performed under supraclavicular brachial plexus block: A randomized controlled study. Egypt. J. Anaesth..

[34] Tharion J.G., Kale S. (2021). Patient Satisfaction Through an Immersive Experience Using a Mobile Phone-Based Head-Mounted Display During Arthroscopic Knee Surgery Under Spinal Anesthesia: A Randomized Clinical Trial. Anesth. Analg..

[35] Ghobadi A., Moradpoor H., Sharini H., Khazaie H., Moradpoor P. (2024). The effect of virtual reality on reducing patients’ anxiety and pain during dental implant surgery. BMC Oral Health.

[36] Gurbuz E., Gurbuz A.A. (2024). Investigation of the effect of virtual reality distraction in patients undergoing mandibular periodontal surgery: A randomized controlled study. J. Esthet. Restor. Dent..

[37] Book F., Goedeke J., Poplawski A., Muensterer O.J. (2020). Access to an online video enhances the consent process, increases knowledge, and decreases anxiety of caregivers with children scheduled for inguinal hernia repair: A randomized controlled study. J. Pediatr. Surg..

[38] Yeo H., Park H. (2023). Benefits of a Single-Session, In-Hospital Preoperative Education Program for Patients Undergoing Ostomy Surgery: A Randomized Controlled Trial. J. Wound Ostomy Cont. Nurs..

[39] Hou H., Li X., Song Y., Ji Y., Sun M., Wang D., Jiao J., Qu J., Gu H. (2023). Effect of interactive, multimedia-based home-initiated education on preoperative anxiety inchildren and their parents: A single-center randomized controlled trial. BMC Anesthesiol..

[40] Güzel N., Yava A., Koyuncu A. (2024). The Effects of Preoperative Video-Assisted Education on Anxiety and Comfort After Breast Cancer Surgery: Nonrandomized Controlled Study. J. PeriAnesthesia Nurs..

[41] Karalar M., Demirbas A., Gercek O., Topal K., Keles I. (2023). Impact of Preoperative Video-Based Education on Anxiety Levels in Patients with Renal Stones Scheduled for Flexible Ureteroscopic Lithotripsy: A Comparative Study Using APAIS and STAI. Med. Sci. Monit. Int. Med. J. Exp. Clin. Res..

[42] van Steenbergen G., van Veghel D., van Lieshout D., Sperwer M., Ter Woorst J., Dekker L. (2022). Effects of Video-Based Patient Education and Consultation on Unplanned Health Care Utilization and Early Recovery After Coronary Artery Bypass Surgery (IMPROV-ED): Randomized Controlled Trial. J. Med. Internet Res..

[43] Lai V.K.W., Ho K.M., Wong W.T., Leung P., Gomersall C.D., Underwood M.J., Joynt G.M., Lee A. (2021). Effect of preoperative education and ICU tour on patient and family satisfaction and anxiety in the intensive care unit after elective cardiac surgery: A randomised controlled trial. BMJ Qual. Saf..

[44] Bozkul G., Karakul A., Düzkaya D.S., Dilşen Ş. (2024). Effect of short film video and video-based education on fear, pain, and satisfaction of children undergoing day surgery. J. Pediatr. Nurs..

[45] Carbó A., Tresandí D., Tril C., Fernández-Rodríguez D., Carrero E. (2024). Usefulness of a virtual reality educational program for reducing preoperative anxiety in children: A randomised, single-centre clinical trial. Eur. J. Anaesthesiol..

[46] Liu S.J., Yen W.J., Chang Y.Z., Ku M.S. (2023). Impact of educational videos on maternal anxiety caused by children’s heart surgery. Nurs. Crit. Care.

[47] Bozdogan Yesilot S., Ciftci H., Ozcelik Z. (2021). The effect of virtual reality on mothers’ anxiety during children’s circumcision: A randomized controlled study. Int. J. Nurs. Pract..

[48] Chiu P.L., Li H., Yap K.Y., Lam K.C., Yip P.R., Wong C.L. (2023). Virtual Reality-Based Intervention to Reduce Preoperative Anxiety in Adults Undergoing Elective Surgery: A Randomized Clinical Trial. JAMA Netw. Open.

[49] Reinders I.M.A., Cremers G.R., van Rooijen S.J., Leemans J.C., Perquin C.W., Geomini P., Maas J.W.M., Bongers M.Y. (2022). The effect of an informative 360-degree virtual reality video on anxiety for women visiting the one-stop clinic for abnormal uterine bleeding: A randomized controlled trial (VISION-trial). Eur. J. Obstet. Gynecol. Reprod. Biol..

[50] Turgut A., Özcan İlçe A., Öztürk H. (2024). The Effect of Immersive Virtual Reality Application on Anxiety, Pain, and Parental Satisfaction in the Perioperative Process of Children: A Randomized Controlled Trial. Pain Manag. Nurs..

[51] Yom K.H., Shriver E.M., Carter K.D., Korn B.S., Kikkawa D.O., Ko A.C. (2021). The Effect of Photographic Visual Aids in Preoperative Patient Counseling in Oculoplastic Surgery. Ophthalmic Plast. Reconstr. Surg..

[52] Omezli M.M., Torul D., Kahveci K. (2020). Does Watching Videos Increase the Perioperative Anxiety in Patients Undergoing Third Molar Surgery? A Randomized Trial. J. Oral Maxillofac. Surg..

[53] Matthyssens L.E., Vanhulle A., Seldenslach L., Vander Stichele G., Coppens M., Van Hoecke E. (2020). A pilot study of the effectiveness of a serious game CliniPup^®^ on perioperative anxiety and pain in children. J. Pediatr. Surg..

[54] Nair T., Choo C.S.C., Abdullah N.S., Lee S., Teo L.L.E., Chen Y., Nah S.A., Chiang L.W. (2021). Home-Initiated-Programme-to-Prepare-for-Operation: Evaluating the effect of an animation video on peri-operative anxiety in children: A randomised controlled trial. Eur. J. Anaesthesiol..

[55] Pedramrazi S., Mohammadabadi A., Rooddehghan Z., Haghani S. (2024). Effectiveness of Peer-Based and Conventional Video Education in Reducing Perioperative Depression and Anxiety Among Coronary Artery Bypass Grafting Patients: A Randomized Controlled Trial. J. PeriAnesthesia Nurs..

[56] Zhang H., Chen W., Wang J., Che G., Huang M. (2024). Real-world study on the application of enhanced recovery after surgery protocol in video-assisted thoracoscopic day surgery for pulmonary nodule resection. BMC Surg..

[57] Fritz Z., Holton R. (2019). Too much medicine: Not enough trust?. J. Med. Ethics.

[58] Nikumb V.B., Banerjee A., Kaur G., Chaudhury S. (2009). Impact of doctor-patient communication on preoperative anxiety: Study at industrial township, Pimpri, Pune. Ind. Psychiatry J..

[59] Tajbakhsh A., Salimi S., Daftarian N., Abtahi D. (2023). Effect of Music During General Anesthesia on Anesthetic Consumption During Vitrectomy Surgery. Adv. Biomed. Res..

[60] Guerrier G., Bernabei F., Lehmann M., Pellegrini M., Giannaccare G., Rothschild P.-R. (2021). Efficacy of Preoperative Music Intervention on Pain and Anxiety in Patients Undergoing Cataract Surgery. Front. Pharmacol..

[61] Loong L.J., Ling K.K., Tai E.L.M., Kueh Y.C., Kuan G., Hussein A. (2022). The Effect of Binaural Beat Audio on Operative Pain and Anxiety in Cataract Surgery under Topical Anaesthesia: A Randomized Controlled Trial. Int. J. Environ. Res. Public Health.

[62] Bürlukkara S., Demir D., Baran Ö. (2024). The effect of music therapy on anxiety and pain scores in patients undergoing retrograde intrarenal surgery (RIRS) under spinal anesthesia: A prospective, randomized controlled clinical trial. Urolithiasis.

[63] Yücel Ş., Küçük Alemdar D. (2024). The effect of listening to music and foot reflexology on nausea, pain and anxiety in children during perioperative period: A randomized controlled study. J. Pediatr. Nurs..

[64] Shukla A., Kaushik N., Hemlata H., Verma R., Gautam S., Singh G.P. (2023). Improvement in Patient Satisfaction and Anxiety With Perioperative Music Therapy in Patients Undergoing Total Abdominal Hysterectomy: A Single-Blind Prospective Study. Cureus.

[65] Giordano F., Giglio M., Sorrentino I., Dell’Olio F., Lorusso P., Massaro M., Tempesta A., Limongelli L., Selicato L., Favia G. (2023). Effect of Preoperative Music Therapy Versus Intravenous Midazolam on Anxiety, Sedation and Stress in Stomatology Surgery: A Randomized Controlled Study. J. Clin. Med..

[66] Chen Y.B., Barnes H., Westbay L., Wolff B., Shannon M., Adams W., Acevedo-Alvarez M., Mueller E.R., Pham T.T. (2021). Preoperative Music Listening in Pelvic Reconstructive Surgery: A Randomized Trial. Female Pelvic Med. Reconstr. Surg..

[67] Koelsch S., Fuermetz J., Sack U., Bauer K., Hohenadel M., Wiegel M., Kaisers U.X., Heinke W. (2011). Effects of Music Listening on Cortisol Levels and Propofol Consumption during Spinal Anesthesia. Front. Psychol..

[68] Azi L., Azi M.L., Viana M.M., Panont A.L.P., Oliveira R.M.F., Sadigursky D., Alencar D.F. (2021). Benefits of intraoperative music on orthopedic surgeries under spinal anesthesia: A randomized clinical trial. Complement. Ther. Med..

[69] Gökçek E., Kaydu A. (2020). The effects of music therapy in patients undergoing septorhinoplasty surgery under general anesthesia. Braz. J. Otorhinolaryngol..

[70] Ezepue C.O., Anyatonwu O.P., Duru C.C., Odini F., Nwachukwu N.Z., Onoh C., Nwachukwu N., Oguonu C.A. (2024). Effects of music on the preoperative and intraoperative anxiety through the assessment of pupil size and vital signs (blood pressure, respiratory, and pulse rates) among cataract surgery patients at UNTH-Enugu. Front. Ophthalmol..

[71] Kavak Akelma F., Altınsoy S., Arslan M.T., Ergil J. (2020). Effect of favorite music on postoperative anxiety and pain. Anaesthesist.

[72] Stahl S.M. (2021). The Amygdala and the Neurobiology of Fear. Stahl’s Essential Psychopharmacology: Neuroscientific Basis and Practical Applications.

[73] Drzymalski D.M., Lumbreras-Marquez M.I., Tsen L.C., Camann W.R., Farber M.K. (2020). The effect of patient-selected or preselected music on anxiety during cesarean delivery: A randomized controlled trial. J. Matern.-Fetal Neonatal Med..

[74] Reynaud D., Bouscaren N., Lenclume V., Boukerrou M. (2021). Comparing the effects of self-selected MUsic versus predetermined music on patient ANXiety prior to gynaecological surgery: The MUANX randomized controlled trial. Trials.

[75] Uğraş G.A., Yıldırım G., Yüksel S., Öztürkçü Y., Kuzdere M., Öztekin S.D. (2018). The effect of different types of music on patients’ preoperative anxiety: A randomized controlled trial. Complement. Ther. Clin. Pract..

[76] Pellicer L.E., Rubio J.L.M., Casañas E., Villar A.C. (2024). Immediate implant placement influenced by musical flow: A prospective randomized controlled clinical trial. BMC Oral Health.

[77] Parodi A., Fodde P., Pellecchia T., Puntoni M., Fracchia E., Mazzella M. (2021). A randomized controlled study examining a novel binaural beat technique for treatment of preoperative anxiety in a group of women undergoing elective caesarean section. J. Psychosom. Obstet. Gynecol..

[78] Demirci H., van der Storm S.L., Huizing N.J., Fräser M., Stufkens S.A.S., Krips R., Kerkhoffs G., Barsom E.Z., Schijven M.P. (2023). Watching a movie or listening to music is effective in managing perioperative anxiety and pain: A randomised controlled trial. Knee Surg. Sports Traumatol. Arthrosc..

[79] Kaur H., Shukla V., Singhal R., Harsh H.K., Pareek R. (2024). The Effect of Intraoperative Music on Sedation, Anxiety, and Hemodynamic Responses among Patients Undergoing Lower Segment Cesarean Section under Spinal Anesthesia. J. Obstet. Anaesth. Crit. Care.

[80] Abdul Hamid M.R., Mansor M.B., Zainal Abidin M.F. (2022). Music therapy for reducing anxiety in patients undergoing total knee replacement surgery under subarachnoid anesthesia. J. Orthop. Surg..

[81] Golitaleb M., Harorani M., Garshasbi M., Akbari M., Jamilian H., Barati N., Habibi D., Hoseini T. (2023). Comparing the Effect of Music and Puzzle-Solving on Anxiety Before Surgery in Children: A Randomized Clinical Trial. Turk. Arch. Pediatr..

[82] Nguyen C.V., Alvin M., Lee C., George D., Gilmore A., Tripi P.A., Liu R.W. (2021). A prospective randomised study on efficacy of music for decreasing preoperative anxiety in children. J. Perioper. Pract..

[83] Wakana K., Kimura Y., Nitta Y., Fujisawa T. (2022). The Effect of Music on Preoperative Anxiety in an Operating Room: A Single-Blind Randomized Controlled Trial. Anesth. Prog..

[84] Setzer W.N. (2009). Essential oils and anxiolytic aromatherapy. Nat. Prod. Commun..

[85] Cooke B., Ernst E. (2000). Aromatherapy: A systematic review. Br. J. Gen. Pract..

[86] Michalak M. (2018). Aromatherapy and methods of applying essential oils. Arch. Physiother. Glob. Res..

[87] Yoo O., Park S.-A. (2023). Anxiety-Reducing Effects of Lavender Essential Oil Inhalation: A Systematic Review. Healthcare.

[88] Wang J.Y., Huang H.Y., Chu W.O., Peng T.R., Lee M.C., Chen S.M., Lee J.A. (2024). Aromatherapy for the prevention of postoperative nausea and vomiting: A systematic review and meta-analysis. Tzu Chi Med. J..

[89] Tang Y., Gong M., Qin X., Su H., Wang Z., Dong H. (2021). The Therapeutic Effect of Aromatherapy on Insomnia: A Meta-Analysis. J. Affect. Disord..

[90] Abdalhai R., Kouchaji C., Alkhatib R. (2024). The effect of aromatherapy with Lavender-Neroli oil and music in management of pediatric dental anxiety: A randomized control trial. BDJ Open.

[91] Franco L., Blanck T.J., Dugan K., Kline R., Shanmugam G., Galotti A., von Bergen Granell A., Wajda M. (2016). Both lavender fleur oil and unscented oil aromatherapy reduce preoperative anxiety in breast surgery patients: A randomized trial. J. Clin. Anesth..

[92] Farzaneh M., Zarean V., Abbasijahromi A., Mohit M., Amirkhani M. (2022). A Randomized Controlled Trial Examining the Effect of Aromatherapy Using the Damask Rose Essential Oil on Pre-operative Anxiety Levels. Nephro-Urol. Mon..

[93] Najafi S., Sajjadi M., Nasirzadeh A., Jeddi H. (2020). The Effect of Rose Aromatherapy on Anxiety Before Abdominal Operation. Intern. Med. Today.

[94] Wotman M., Levinger J., Leung L., Kallush A., Mauer E., Kacker A. (2017). The Efficacy of Lavender Aromatherapy in Reducing Preoperative Anxiety in Ambulatory Surgery Patients Undergoing Procedures in General Otolaryngology. Laryngoscope Investig. Otolaryngol..

[95] Nirmala K., Kamatham R. (2021). Effect of Aromatherapy on Dental Anxiety and Pain in Children Undergoing Local Anesthetic Administrations: A Randomized Clinical Trial. J. Caring Sci..

[96] Rahman R., Vasu Dewan M., Sayed Masri S., Mokhtar M., Abdullah F., Md Nor N. (2024). Lavender aromatherapy: Its effect on preoperative anxiety and propofol requirement for anesthesia. Anaesth. Pain Intensive Care.

[97] Rigon M.R., Donatello N.N., Volpato L.K., Piovezan A.P. (2021). The Use of Essential Oils in Pediatric Care: An Integrative Review. Ann. Pediatr. Child. Health.

[98] Abbasijahromi A., Hojati H., Nikooei S., Jahromi H.K., Dowlatkhah H.R., Zarean V., Farzaneh M., Kalavani A. (2020). Compare the effect of aromatherapy using lavender and Damask rose essential oils on the level of anxiety and severity of pain following C-section: A double-blinded randomized clinical trial. J. Complement. Integr. Med..

[99] Bahrami F., Hanifi N., Mardani A. (2024). Comparison of the Effects of Aromatherapy With Damask Rose and Chamomile Essential Oil on Preoperative Pain and Anxiety in Emergency Orthopedic Surgery: A Randomized Controlled Trial. J. PeriAnesthesia Nurs..

[100] Bozkurt P., Vural Ç. (2019). Effect of Lavender Oil Inhalation on Reducing Presurgical Anxiety in Orthognathic Surgery Patients. J. Oral. Maxillofac. Surg..

[101] Fazlollahpour-Rokni F., Shorofi S.A., Mousavinasab N., Ghafari R., Esmaeili R. (2019). The effect of inhalation aromatherapy with rose essential oil on the anxiety of patients undergoing coronary artery bypass graft surgery. Complement. Ther. Clin. Pract..

[102] Qu J., Shou C., He X., Wang Q., Fang Y. (2024). Analysis of acupoint massage combined with touch on relieving anxiety and pain in patients with oral implant surgery. World J. Psychiatry.

[103] Çavdar A.U., Yılmaz E., Baydur H. (2020). The Effect of Hand Massage Before Cataract Surgery on Patient Anxiety and Comfort: A Randomized Controlled Study. J. PeriAnesthesia Nurs..

[104] Dilaveri C.A., Croghan I.T., Mallory M.J., Dion L.J., Fischer K.M., Schroeder D.R., Martinez-Jorge J., Nguyen M.-D.T., Fokken S.C., Bauer B.A. (2020). Massage Compared with Massage Plus Acupuncture for Breast Cancer Patients Undergoing Reconstructive Surgery. J. Altern. Complement. Med..

[105] Ren N., Yang G., Ren X., Li L. (2021). Effects of foot massage on relieving pain, anxiety and improving quality of life of patients undergone a cervical spine surgery. Health Qual. Life Outcomes.

[106] Gensic M.E., Smith B.R., LaBarbera D.M. (2017). The effects of effleurage hand massage on anxiety and pain in patients undergoing chemotherapy. JAAPA.

[107] Ni C.-H., Wei L., Wu C.-C., Lin C.-H., Chou P.-Y., Chuang Y.-H., Kao C.-C. (2021). Machine-Based Hand Massage Ameliorates Preoperative Anxiety in Patients Awaiting Ambulatory Surgery. J. Nurs. Res..

[108] Li Z., Bauer B., Aaberg M., Pool S., Van Rooy K., Schroeder D., Finney R. (2021). Benefits of hand massage on anxiety in preoperative outpatient: A quasi-experimental study with pre- and post-tests. Explore.

[109] Farmahini Farahani M., Noruzi Zamenjani M., Nasiri M., Shamsikhani S., Purfarzad Z., Harorani M. (2020). Effects of Extremity Massage on Preoperative Anxiety: A Three-Arm Randomized Controlled Clinical Trial on Phacoemulsification Candidates. J. PeriAnesthesia Nurs..

[110] Saltan A., Mert S., Topbaş Ö., Aksu B. (2024). The investigation of effect on foot plantar massage on functional recovery in older adults with general surgery, randomized clinical trial. Aging Clin. Exp. Res..

[111] Mirhosseini S., Abbasi A., Norouzi N., Mobaraki F., Basirinezhad M.H., Mohammadpourhodki R. (2021). Effect of aromatherapy massage by orange essential oil on post-cesarean anxiety: A randomized clinical trial. J. Complement. Integr. Med..

[112] Kain Z.N., Mayes L.C., Bell C., Weisman S., Hofstadter M.B., Rimar S. (1997). Premedication in the United States: A Status Report. Anesth. Analg..

[113] Dubovsky S.L., Marshall D. (2022). Benzodiazepines Remain Important Therapeutic Options in Psychiatric Practice. Psychother. Psychosom..

[114] Stahl S.M. (1998). Mechanism of action of serotonin selective reuptake inhibitors: Serotonin receptors and pathways mediate therapeutic effects and side effects. J. Affect. Disord..

[115] Constable P.A., Al-Dasooqi D., Bruce R., Prem-Senthil M. (2022). A Review of Ocular Complications Associated with Medications Used for Anxiety, Depression, and Stress. Clin. Optom..

[116] Uçar H.K., Arhan E., Serdaroğlu A., Aydın K., Kazancıoğlu A., Akkuzu E., Kalkan G. (2018). First Description of QTc Prolongation Associated With Clonazepam Overdose in a Pediatric Patient. Am. J. Ther..

[117] Schlit A.F., Delaunois A., Colomar A., Claudio B., Cariolato L., Boev R., Valentin J.P., Peters C., Sloan V.S., Bentz J.W.G. (2017). Risk of QT prolongation and torsade de pointes associated with exposure to hydroxyzine: Re-evaluation of an established drug. Pharmacol. Res. Perspect..

[118] Ortiz de Landaluce L., Carbonell P., Asensio C., Escoda N., López P., Laporte J.R. (2018). Gabapentin and Pregabalin and Risk of Atrial Fibrillation in the Elderly: A Population-Based Cohort Study in an Electronic Prescription Database. Drug Saf..

[119] Griffin C.E., Kaye A.M., Bueno F.R., Kaye A.D. (2013). Benzodiazepine pharmacology and central nervous system-mediated effects. Ochsner J..

[120] Euteneuer F., Kampmann S., Rienmüller S., Salzmann S., Rüsch D. (2022). Patients’ desires for anxiolytic premedication—An observational study in adults undergoing elective surgery. BMC Psychiatry.

[121] Kaplan K., Hunsberger H.C. (2023). Benzodiazepine-induced anterograde amnesia: Detrimental side effect to novel study tool. Front. Pharmacol..

[122] Hsu T.W., Chen H.M., Chen T.Y., Chu C.S., Pan C.C. (2021). The Association between Use of Benzodiazepine Receptor Agonists and the Risk of Obstructive Sleep Apnea: A Nationwide Population-Based Nested Case-Control Study. Int. J. Environ. Res. Public Health.

[123] The Royal Australian College of General Practitioners Prescribing Drugs of Dependence in General Practice, Part B—Benzodiazepines. https://www.racgp.org.au/clinical-resources/clinical-guidelines/key-racgp-guidelines/view-all-racgp-guidelines/drugs-of-dependence/part-b.

[124] Stahl S.M. (2021). Serotonin and Anxiety. Stahl’s Essential Psychopharmacology: Neuroscientific Basis and Practical Applications.

[125] Taylor M.J., Freemantle N., Geddes J.R., Bhagwagar Z. (2006). Early onset of selective serotonin reuptake inhibitor antidepressant action: Systematic review and meta-analysis. Arch. Gen. Psychiatry.

[126] Longo L.P., Johnson B. (2000). Addiction: Part I. Benzodiazepines--side effects, abuse risk and alternatives. Am. Fam. Physician.

[127] Agbayani C.J.G., Fortier M.A., Kain Z.N. (2020). Non-pharmacological methods of reducing perioperative anxiety in children. BJA Educ..

[128] Rogula S. (2025). Pharmacological and Nonpharmacological Methods for Reducing Perioperative Anxiety. https://BioRender.com/z62x890.

[129] Koffel E., Hagedorn H. (2020). Provider perspectives of implementation of an evidence-based insomnia treatment in Veterans Affairs (VA) primary care: Barriers, existing strategies, and future directions. Implement. Sci. Commun..

